# Computational-approach understanding the structure-function prophecy of Fibrinolytic Protease RFEA1 from *Bacillus cereus* RSA1

**DOI:** 10.7717/peerj.11570

**Published:** 2021-06-04

**Authors:** Chhavi Sharma, Arti Nigam, Rajni Singh

**Affiliations:** 1 Amity Institute of Microbial Technology, Amity University Uttar Pradesh, Noida, India; 2 Department of Microbiology, Institute of Home Economics, Delhi University South Campus, Delhi, India

**Keywords:** Cardiovascular diseases, Fibrinolytic enzyme RFEA1, Computer aided structure-function prediction, Molecular docking, PATCH DOCK, FIRE DOCK

## Abstract

Microbial fibrinolytic proteases are therapeutic enzymes responsible to ameliorate thrombosis, a fatal cardiac-disorder which effectuates due to excessive fibrin accumulation in blood vessels. Inadequacies such as low fibrin specificity, lethal after-effects and short life-span of available fibrinolytic enzymes stimulates an intensive hunt for novel, efficient and safe substitutes. Therefore, we herewith suggest a novel and potent fibrinolytic enzyme RFEA1 from *Bacillus cereus* RSA1 (MK288105). Although, attributes such as in-vitro purification, characterization and thrombolytic potential of RFEA1 were successfully accomplished in our previous study. However, it is known that structure-function traits and mode of action significantly aid to commercialization of an enzyme. Also, predicting structural model of a protein from its amino acid sequence is challenging in computational biology owing to intricacy of energy functions and inspection of vast conformational space. Our present study thus reports In-silico structural-functional analysis of RFEA1. Sequence based modelling approaches such as—Iterative threading ASSEmbly Refinement (I-TASSER), SWISS-MODEL, RaptorX and Protein Homology/analogY Recognition Engine V 2.0 (Phyre2) were employed to model three-dimensional structure of RFEA1 and the modelled RFEA1 was validated by structural analysis and verification server (SAVES v6.0). The modelled crystal structure revealed the presence of high affinity Ca1 binding site, associated with hydrogen bonds at Asp^147^, Leu^181^, Ile^185^ and Val^187^residues. RFEA1 is structurally analogous to Subtilisin E from *Bacillus subtilis* 168. Molecular docking analysis using PATCH DOCK and FIRE DOCK servers was performed to understand the interaction of RFEA1 with substrate fibrin. Strong RFEA1-fibrin interaction was observed with high binding affinity (−21.36 kcal/mol), indicating significant fibrinolytic activity and specificity of enzyme RFEA1. Overall, the computational research suggests that RFEA1 is a subtilisin-like serine endopeptidase with proteolytic potential, involved in thrombus hydrolysis.

## Introduction

Fibrinolytic enzymes are peptide hydrolases (EC 3.4) accountable for blood clot (thrombus) dissolution and subsequently reinstate ideal vascular architecture (Ali & Bavisetty, 2020; Krishnamurthy, Belur & Subramanya, 2018; Kotb, 2013). Thrombin, a coagulation protein, mediates cleavage of a glycoprotein fibrinogen into fibrin monomers which after polymerization forms blood clot and obstruct lethal haemorrhage (Göbel et al., 2018; Weisel, 2005; Fuss, Palmaz & Sprague, 2001; Mosesson, 1999). Under natural physiological conditions, fibrin deposition is hydrolysed by plasmin, effectuating clot dissolution to avoid thrombotic complications (Sidelmann et al., 2000). However, this constant dynamic equilibrium is disturbed due to numerous pathophysiological shambles leading to life threatening cardiovascular thrombosis (Göbel et al., 2018; Sharma et al., 2020). The World Health Organization delineated that cardiovascular diseases causes 17.9 million deaths per year and projects 31% of global mortality (https://www.who.int/health-topics/cardiovascular-diseases#tab=tab_1).

Notably, anticoagulants and antiplatelet drugs such as apixaban, warfarin, dabigatran, aspirin or dipyridamole have been employed for thrombus hydrolysis but are highly expensive and leave undesirable after-effects such as haemorrhage, esophagitis, gastrointestinal discomfort and alopecia etc. (Thachil, 2016; Watras, Patel & Arya, 2016; Yoshihide, Eri & Hajime, 2019). Thrombolysis therapy involving the use of microbial fibrinolytic enzymes is thus preferably used to combat thrombosis. Extensive industrial and therapeutic applicability of fibrinolytic enzymes has increased interest in understanding their mechanism of action and structure-function properties (Bora et al., 2017). Fibrinolytic enzymes based on their catalytic mechanism are classified as serine proteases (EC. 3.4.21), metalloproteases (EC 3.4.24) and serine metalloproteases (Raju & Divakar, 2014). Serine proteases cleave peptide bonds, where serine is present as nucleophilic amino acid at enzyme’s active site (Page & Cera, 2008) while metalloproteases necessitate the administration of metal ions (Zn^2+^, Ca^2+^, Co^2+^, Mg^2+^etc.) to perform varied biological functions such as substrate recognition/binding, electron transfer and catalysis (Chen et al., 2019). The fibrinolytic enzyme of the third category, i.e serine metalloprotease exhibits both serine and metalloprotease properties (Peng, Yang & Zhang, 2005).

Additionally, research-oriented pharmaceutical industry progressively necessitates 3D protein structural and functional elucidation using bioinformatic molecular modelling tools (Ferreira et al., 2015). Henceforth, structure based functional characterization of a protein is preeminent goal in biological sciences (Manjasetty et al., 2012). Also, biochemical/cellular processes are mainly controlled by intermolecular protein-ligand interactions with efficient physiological substrate and inhibitor/activator specification. Such computer-aided interaction is carried out with two independent three-dimensional (3D) crystallized ligand co-ordinates to obtain and examine the bound structure model (Bora et al., 2017).

In this work, we thus investigated structure-function attributes of fibrinolytic protease RFEA1 via an In-silico approach. Molecular docking was performed to study RFEA1-substrate interaction. Besides, multiple sequence alignment and sequence logos were generated to study comparative analysis with similar sequences and recognize the conserved motifs.

## Materials & Methods

### Fibrinolytic protease RFEA1 production and identification

Fibrinolytic protease producing strain *Bacillus cereus* RSA1 (NCBI Accession number MK288105) was isolated from soil samples, procured from numerous garbage dumps of Noida (U.P, India). Bergey’s brochure for identification of bacteriology and 16S rDNA sequencing was employed for identification of the strain. The fibrinolytic protease production was statistically optimized in our previous study (Plackett Burman Design and Central Composite Design using Design Expert^®^10.0.8.0, Stat-Ease Inc., Minneapolis, MN, USA) which comprised of peptone (10 g/L), yeast extract (5 g/L), and glucose (0.5 g/L) with final pH 8.0. The cultures were incubated for 24 h at 37 °C with 1% inoculum and agitation rate of 120 rpm. The enzyme was purified as per our standardized protocol in previous study, employing chilled ethanol precipitation and gel filtration chromatography–Sephadex G75 column (50 × 15 mm; Sigma Aldrich, St. Louis, MO, USA), and identified through MALDI-TOF Mass Spectroscopy (Sharma et al., 2020). Further, statistical compositional amino acid outcome of protein RFEA1 was determined using statistical tool SAP Application Performance Standard (SAPS: http://www.ebi.ac.uk/Tools/seqstats/saps/). SAPS utilize FASTA organized amino acid sequence of individual protein and investigates its composition, repetitive structure, charge dispersion, dividing and multiple periodicity (Brendel et al., 1992).

### In-silico structural modeling and validation of RFEA1

The structural modelling of RFEA1 was performed using bioinformatic webservers such as I-TASSER (https://zhanglab.ccmb.med.umich.edu/I-TASSER/) (Roy, Kucukural & Zhang, 2010; Yang et al., 2015; Yang & Zhang, 2015), SWISS-MODEL (https://swissmodel.expasy.org/) (Waterhouse et al., 2018), RaptorX (http://raptorx.uchicago.edu/) (Xu, Mcpartlon & Li, 2020; Xu, 2019; Xu & Wang, 2019; Wang et al., 2017a; Wang, Sun & Xu, 2018; Wang et al., 2017b) and Phyre2 (http://www.sbg.bio.ic.ac.uk/phyre2) (Kelley et al., 2015). The modelled RFEA1 structures were validated by structural analysis and verification server (SAVES v6.0, https://servicesn.mbi.ucla.edu/SAVES/). SAVES metaserver runs six different programs for validating the submitted protein structure. We have used Ramachandran Plot (RC plot) (Carugo & Djinovic-Carugo, 2013), ERRAT (Colovos & Yeates, 1993) and Verify3D (Bowie, Lüthy & Eisenberg, 1991; Lüthy, Bowie & Eisenberg, 1992) for the same.

### Analysis of Ca1 binding site in RFEA1

Ligplot^+^ v.2.2 (https://www.ebi.ac.uk/thornton-srv/software/LigPlus/) (Laskowski & Swindells, 2011) was employed for detection and visualization of the metal cation (Ca1) binding sites in modelled RFEA1 structure.

### Determination of structural analogs of RFEA1

TM-align (https://zhanglab.ccmb.med.umich.edu/TM-align/), an algorithm for protein structure alignment and comparison was employed to identify structural analogs of validated RFEA1 model in PDB library. TM-align employ heuristic dynamic programming iterations to produce residue to residue alignment for two protein structures of unfamiliar uniformity. The algorithm reports top 10 proteins from PDB with closest structural similarity and provides TM-score (ranges from 0 to 1) determining highest degree of structural similarity. Higher the TM-score value, better and perfect is the structural match (Zhang & Skolnick, 2005).

### Prediction of enzyme commission numbers and gene ontology terms of RFEA1

COFACTOR (https://zhanglab.ccmb.med.umich.edu/COFACTOR/) online meta-server was employed for reporting molecular and biological functional annotations of RFEA1. COFACTOR subjects the query BioLiP to identify functional insights such as Enzyme Commission number (EC) and Gene Ontology (GO). COFACTOR provides detailed insights about GO from UniProt-GOA and STRING databases. Thus, structure-based function of RFEA1 was predicted using COFACTOR (Roy, Yang & Zhang, 2012; Zhang, Freddolino & Zhang, 2017), wherein the server provides C-Score values (range 0 to 1), where higher score indicates high reliability of each outcome.

### Molecular docking

PATCH DOCK v1.3 (http://bioinfo3d.cs.tau.ac.il/PatchDock) (Duhovny, Nussinov & Wolfson, 2002; Schneidman-Duhovny et al., 2005) was employed for conducting docking analysis of RFEA1 with protein fibrin (PDB ID 2HLO). The structure of fibrin in PDB format was obtained from protein data bank (https://www.rcsb.org/) for the study. The modelled structure of RFEA1 by Phyre2 server was used as receptor molecule. PATCH DOCK server is a geometry-based docking algorithm based on shape complementarity principles and operates RMSD clustering to eradicate superfluous solutions. In our study, we have used default RMSD value of 4 Å. PATCH DOCK protein-protein complex results were further refined using Fast Interaction REfinement in molecular DOCKing **(**FIRE DOCK), (http://bioinfo3d.cs.tau.ac.il/FireDock/php.php) (Andrusier, Nussinov & Wolfson, 2007; Mashiach et al., 2008). The output file of docked complex was visualized with UCSF Chimera 1.15rc (https://www.cgl.ucsf.edu/chimera/) (Pettersen et al., 2004) whereas 2D plot was analyzed and deduced using Ligplot^+^ v.2.2.

### In-vitro validation

In-vitro validation and comparative analysis (with our previous research) of the bio-informatics outcome was performed to draw conclusive decisions. The effect of calcium ion on efficacy and thermostability of RFEA1 was studied and confirmed in-vitro. RFEA1 was purified as per our standardized protocol in previous study, employing chilled ethanol precipitation and gel filtration chromatography—Sephadex G75 column (50 × 15 mm; Sigma Aldrich, St. Louis, MO, USA) (Sharma et al., 2020). The enzyme was incubated for 2 h at 37 °C with different concentrations of CaCl_2_ (0.5–3.0 mM) and then tested for fibrin hydrolysis. The fibrinolytic activity assay was performed according to Sharma et al. (2020). Further, in another set of experiments we have used 2 mM of CaCl_2_ and stability of RFEA1 was tested at different temperatures (20–80 °C). Solution with no CaCl_2_ was considered control. All the experiments were performed in triplicates and statistically analyzed. The In-silico interaction of RFEA1 with substrate (fibrin) was compared with our previous in-vitro study.

### Identification of conserved domain motifs of RFEA1

The homologous enzymes of RFEA1 were explored through protein sequence similarity search tool: PSI-BLAST (https://www.ebi.ac.uk/Tools/sss/psiblast/). The multiple sequence alignment for the best 10 homologous hits obtained from PSI-BLAST was performed using CLUSTALW (https://www.ebi.ac.uk/Tools/msa/clustalo/) (Madeira et al., 2019) and visualized with JalView version 2.11.1.0 (Waterhouse et al., 2009). Further, WebLogo 3.7.4 program (http://weblogo.threeplusone.com/) was used to generate sequence logos for a clear alignment of identified conserved domains (Crooks et al., 2004; Schneider & Stephens, 1990).

## Results

### Procured amino acid sequence of fibrinolytic protease RFEA1 and SAP application performance standard analysis

The protein sequence of RFEA1 derived through MALDI-TOF mass spectrometric analysis in our previous study with Mr 39,483 Da and sequence score of 381 amino acids is mentioned underneath (Sharma et al., 2020).

MRSKKLWISLLFALTLIFTMAFSNMSAQAAGKSSTEKKYIVGFKQTMSAMSSAKKKDVISEKGGKVQKQFKYVNAAAATLDEKAVKELKKDPSVAYVEEDHIAHEYAQSVPYGISQIKAPALHSQGYTGSNVKVAVIDSGIDSSHPDLNVRGGASFVPSETNPYQDGSSHGTHVAGTIAALNNSIGVLGVAPSASLYAVKVLDSTGSGQYSWIINGIEWAISNNMDVINMSLGGPTGSTALKTVVDKAVSSGIVVAAAAGNEGSSGSTSTVGYPAKYPSTIAVGAVNSSNQRASFSSVGSELDVMAPGVSIQSTLPGGTYGAYNGTSMATPHVAGAAALILSKHPTWTNAQVRDRLESTATYLGNSFYYGKGLINVQAAAQ

Bioinformatic tool: SAPS confirmed the molecular weight of RFEA1 (39.5 kDa) and specified statistical compositional outcome of the protein RFEA1 as:

A : 48 (12.6%); D : 13 (3.4%); E : 12 (3.1%); F : 8 (2.1%); G: 37 (9.7%); H : 8 (2.1%); I : 21 (5.5%); K : 25 (6.6%); L : 22 (5.8%); M : 9 (2.4%); N : 18 (4.7%); P : 14 (3.7%); Q : 14 (3.7%); R- : 5 (1.3%); S+ : 50 (13.1%); T : 24 (6.3%); V : 33 (8.7%); W : 4 (1.0%); Y : 16 (4.2%). The results indicate that RFEA1 is a serine (13.1%) and alanine (12.6%) rich protein.

### Structural modelling of RFEA1 using multiple servers

**Figure 1:**
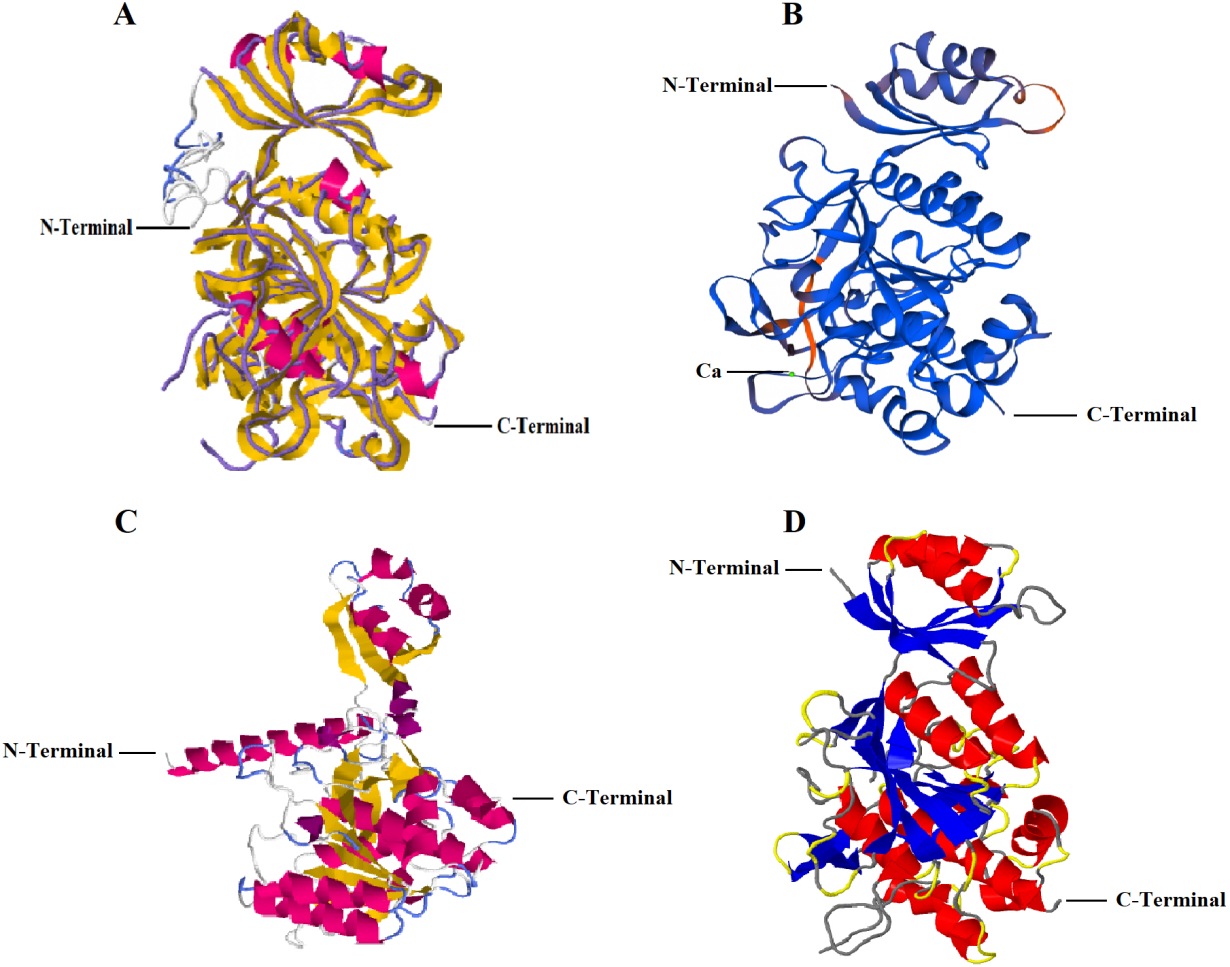
Structural modelling of RFEA1 using multiple servers. (A) I-TASSER modelled RFEA1 showing C-Score: −0.16, estimated TM-score =0.69 ± 0.12 and estimated RMSD =7.1 ± 4.1 Å. Superposition of template protein (3whiA) and query protein (RFEA1) is presented in purple backbone trace and cartoon style, respectively. Magenta colour in cartoon model here indicates alpha-helices and yellow colour signify beta-sheets. (B) RFEA1 modelling by SWISS-MODEL showing calcium binding site (Ca1) with GMQE score: 0.83 and QMEAN score: −0.82. Model is presented in colours based on QMEAN quality score for clear conception of well modelled (blue) and poorly modelled (orange) regions. (C) Cartoon style modelled RFEA1 by RAPTORX with estimated RMSD =6.9122 Å. (D) RFEA1 model generated by Phyre2 tool, with a rainbow colour-coded confidence (blue for minimum and red with maximum confidence).

I-TASSER predicted five models with C-score: −0.16 (Model 1), −1.33 (Model 2), −2.42 (Model 3), −3.24 (Model 4), −0.86 (Model 5). C-score ranges between −5 and 2 where higher conviction is controlled by higher C-score. Thus, first of the five projected models by I-TASSER online server i.e., Model 1 with highest confidence score was selected (Fig. 1A). The analysis further revealed that RFEA1 exhibited maximum sequence identity (99%) with crystal structure of unautoprocessed form of IS1-inserted Pro-subtilisin E template (IS1-ProS221A) (PDB ID: 3whi.1.A) and henceforth structural imposition of RFEA1 with template protein (3whiA) is displayed in Fig. 1A. The estimated template modeling (TM) score (ranges between 0 and 1) is a projected scale for evaluating the structural resemblance between two structures whereas estimated root-mean-square deviation (RMSD) signify an average distance of all residue pairs of 3D model and its experimental structure (Zhang & Skolnick, 2004; Xu & Zhang, 2010). A higher TM-Score specify superior structural match while smaller RMSD value signifies good quality of model. TM-score of Model 1 is 0.69 ± 0.12 and RMSD is 7.1 ± 4.1 Å, which confirms precise topology. The outcome also revealed that projected model possessed single chain comprising ten alpha-helices and sixteen beta-strands. Fig. 1B represents structural modeling of RFEA1 using second server SWISS-MODEL. According to the predicted GMQE (0.83) and QMEAN (−0.82), RFEA1 showed maximum identification (99.15%) to the ‘3whi’ chain A of pro-subtilisin E of *Bacillus subtilis*. The modelled RFEA1 presented calcium binding site (Ca1) as a part of its structure and five residues (Asp^147^, Leu^181^, Asn^183^, Ile^185^ and Val^187^) were found within 4 Å for ligand contacts with chain A. Similarly, RFEA1 structural modelling was performed using RaptorX server (Fig. 1C). The tool predicted five models with estimated RMSD (Å): 6.9122 (Model 1), 8.9796 (Model 2), 8.3941 (Model 3), 8.7051 (Model 4) and 10.419 (Model 5). Model 1 with lowest RMSD value was selected and further analysed. The selected model constituted: Strand: 20.5%, Alpha Helix: 32.8%, 3_10_ Helix: 2.9% and Other: 43.8%. Another server, Phyre2 was employed to generate RFEA1 3D model (Fig. 1D). This server projected model with 100% confidence and 99% identity with template ‘3whiA’. Alpha helix (29%), beta strand (25%) and TM helix (4%) were present in the predicted model. Even though there is high similarity between RFEA1 and template 3whiA, the analysis of unprocessed structures through model-template alignment (Fig. S1) suggests that a large segment of 29 residues from position 1–29 (Met^1^–Ala^29^) is lacking in template with respect to RFEA1 while a segment of 13 residues from position 78–90 (Gly^78^–Pro^90^) is lacking in enzyme RFEA1 with respect to template. Furthermore, the alignment clearly indicates huge dissimilarity in the positions of amino acid residues in both RFEA1 and template.

### Validation of the predicted 3D structures of RFEA1

**Figure 2:**
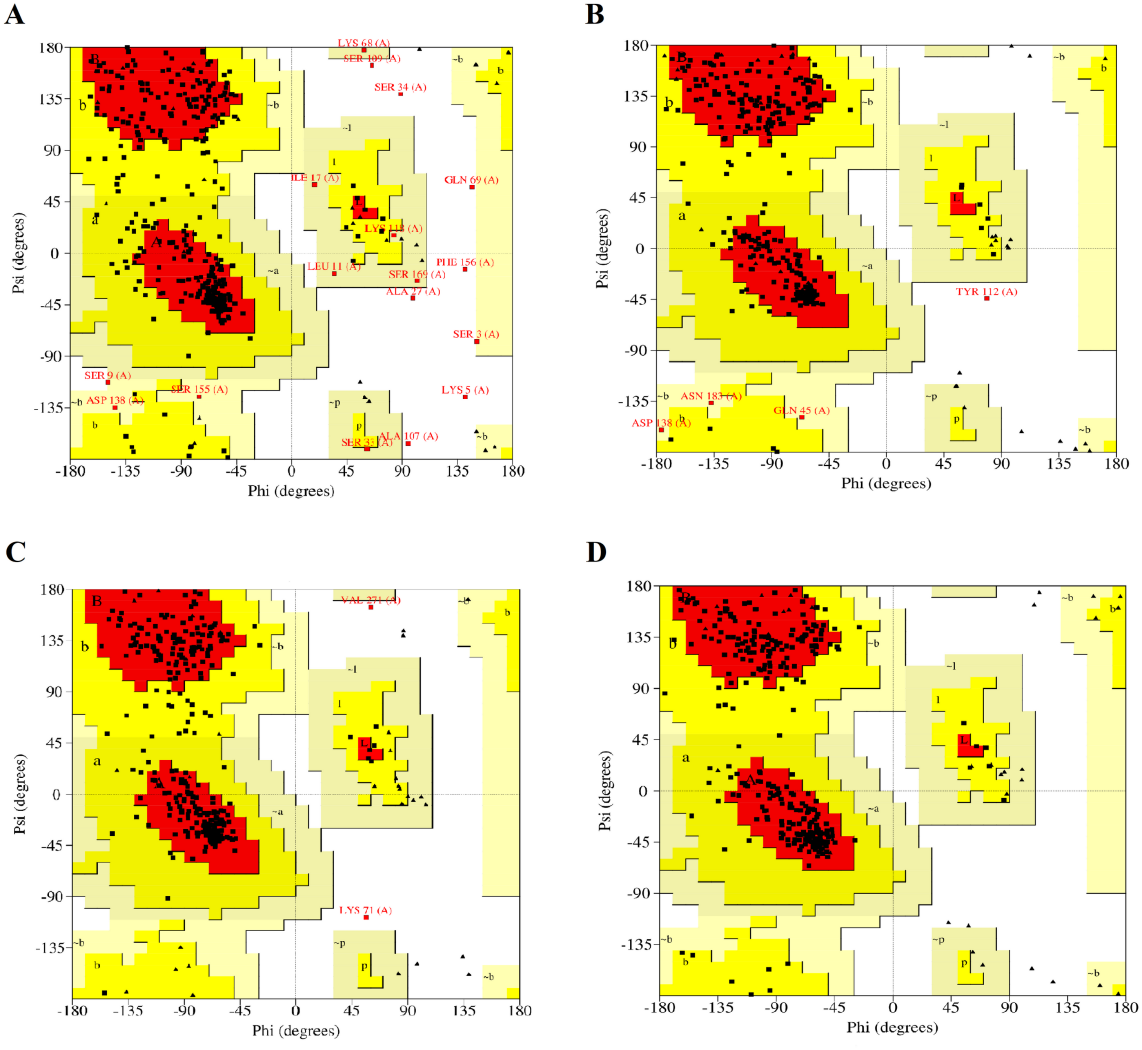
Ramachandran plots for the modelled four structures of RFEA1. Residues present in favoured, allowed and disallowed regions are shown with red, yellow and white colour demarcation, correspondingly. (A) I-TASSER modelled structure. (B) SWISS-MODEL modelled structure. (C) RAPTORX modelled structure. (D) Phyre2 modelled structure.

The modelled RFEA1 structures were further validated through online SAVES v6.0 scrutinizing RC plot (Fig. 2), ERRAT (Fig. S2) and Verify3D (Fig. S3). RC plot for modeled structures of RFEA1 by I-TASSER, SWISS-MODEL, RaptorX and Phyre2 servers showed 72.90, 86.10, 87.80 and 84.60% of residues in most favored region with 2.10, 0.30, 0.60 and 0.00% residues in disallowed regions, respectively (Figs. 2A, 2B, 2C & 2D). Table 1 reveals validation statistics which indicated that Phyre2 server predicted better RFEA1 model than other servers, with maximum (100%) of residues in acceptable region and no (0.00%) residues in disallowed region of RC plot, overall quality factor of 89.64 and 99.42% residues with averaged 3D-ID score >=0.2. Henceforth, validation scores suggest that Phyre2 modelled RFEA1 can be used for further structure-based molecular docking analysis.

**Table 1:** Ramachandran plot statistics, ERRAT and Verify3D scores for RFEA1 generated models using different servers.

	**MF regions**^a^**(%)**	**AA regions**^b^**(%)**	**GA regions**^c^**(%)**	**DA regions**^d^**(%)**	**ERRAT score**^e^**(%)**	**Verify 3D score**^f^**(%)**
I-TASSER	72.90	22.00	3.00	2.10	79.62	88.98
SWISS-MODEL	86.10	12.50	1.00	0.30	92.21	97.98
RAPTORX	87.80	11.60	0.00	0.60	88.20	88.19
Phyre2	84.60	15.40	0.00	0.00	89.64	99.42

**Notes.**

a Ramachandran plot: Residues in most favoured regions [A, B, L].

b Ramachandran plot: Residues in additional allowed regions [a, b, l, p].

c Ramachandran plot: Residues in generously allowed regions [∼a, ∼b, ∼l, ∼p].

d Ramachandran plot: Residues in disallowed regions.

e Overall quality factor generated by ERRAT server.

f Averaged 3D-1D score generated by Verify3D server.

### Assessment of predicted high-affinity calcium binding site in RFEA1

**Figure 3:**
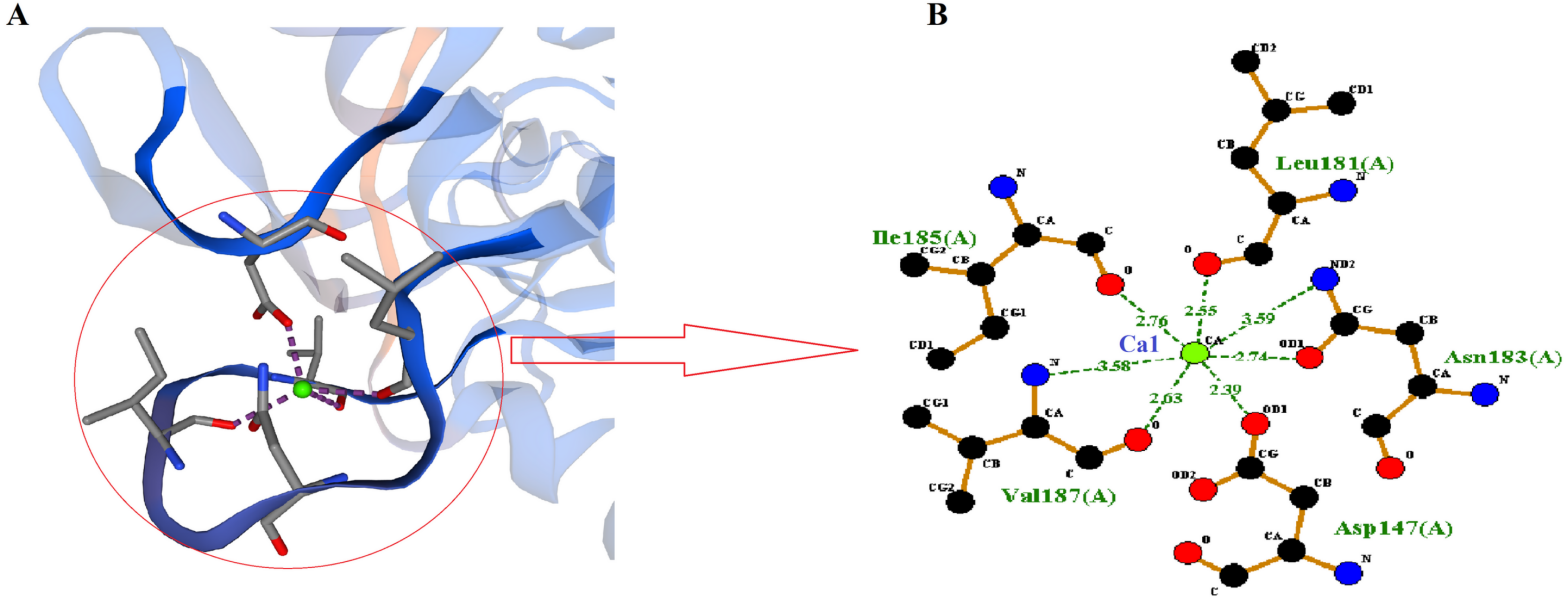
Calcium-binding sites (Ca1) of RFEA1. (A) SWISS-MODEL predicted Ca1 binding sites of RFEA1. (B) RFEA1-Ca1 2D interaction showing the interacting residues (Asp^147^, Leu^181^, Ile^185^ and Val^187^) along with distance between the Ca1 ion and target atom. Green ball in both the diagrams indicates Ca1.

SWISS-MODEL predicted Ca1 binding site of RFEA1 which was analyzed using Ligplot tool. Ligplot v.2.2. analyzed the interaction of Ca1 with chain A (Complex: Ca, tetrahedral) of RFEA1 with residues Asp^147^, Leu^181^, Ile^185^ and Val^187^. Figure 3 presents the 2D view of Ca1 binding sites of RFEA1 along with the distance (Å) between metal and target atom. The Ca1 site of RFEA1 homolog ‘3whi’ was found as ‘Complex: Ca, trigonal.bipyramidal’ with dissimilar amino acid residue positions (in comparison to RFEA1) in addition to radius between Ca1 and target atom at –Asp^131^ (2.18 Å), Asp^131^ (2.57 Å), Leu^165^ (2.23 Å), Ile^169^ (2.27 Å) and Val^171^ (2.43 Å). According to the modelled structure of AprE176, residues Gly^169^, Tyr^171^ and Val^174^ were located within distance of 3.0 Å from Ca ion. Also, for M179, the hydroxyl group of Thr^176^ was located closely (2.9 Å) to Ca ion (Jeong et al., 2015). Several other *Bacillus* enzymes (BPN and Carlsberg) with fibrinolytic potential have been reported with Ca1 and Ca2 sites (Bryan et al., 1992; McPhalen & James, 1988). Furthermore, calcium binding sites (Ca1) Asp^41^, Leu^75^, Ile^79^ and Val^81^ were present in crystal structure of subtilisin-propeptide complex (PDB ID: 1scj) (Jain et al., 1998), complex between subtilisin from a mesophilic bacterium and leech inhibitor eglin-c (PDB ID: 1mee) (Dauter et al., 1991) and crystal structure of nattokinase from *Bacillus subtilis* natto (PDB ID: 4dww) (Yanagisawa et al., 2010). The comparison of RFEA1 with different available crystallographic structures of homologous therapeutic enzymes for Ca1 binding residues is detailed in Table 2.

**Table 2:** Comparative analysis of RFEA1 calcium binding site with homologous templates identified by SWISS-MODEL.

					Co-ordinating residues with chain A of homologous templates	
**Name**	**Identity (%)**		**Sequence similarity**	**Coverage**		**GMQE**		**Ca1**	**Ca2**
RFEA1	–		–	–		0.830		Asp^147^, Leu^181^, Ile^185^ and Val^187^	–
3whi	99.148		0.601	0.924		0.881		Asp^131^, Leu^165^, Ile^169^ and Val^171^	–
5gl8	99.636		0.603	0.722		0.686		–	–
1scj	98.909		0.601	0.722		0.685		Asp^41^, Leu^75^, Ile^79^ and Val^81^	Ala^169^, Tyr^171^, Thr^174^ and H_2_O.1
1mee	98.909		0.601	0.722		0.685		Asp^41^, Leu^75^, Ile^79^ and Val^81^	Ala^169^, Tyr^171^, Thr^174^ and H_2_O.53
6o44	98.545		0.600	0.722		0.686		Asp^41^, Leu^75^, Ile^79^ and Val^81^	–
1yjb	86.909		0.565	0.722		0.673		Asp^41^, Leu^75^, Ile^79^ and Val^81^	Ala^169^, Tyr^171^, Val^174^ and H_2_O.1
1bh6	71.795		0.511	0.717		0.640		Asp^41^, Leu^74^, Thr^78^ and Val^80^	–

### Identification of structural analogs of RFEA1 in Protein Data Bank

The top 10 TM-align identified structural analogs of RFEA1 are detailed in Table 3, in which the first model (Rank 1) with PDB hit: 3whiA holds highest TM-score 0.905. RMSD, IDEN and Cov score of the homolog were 0.81, 0.983 and 0.911 respectively, which designates an acceptable outcome. ‘3whiA’ was observed to be the crystal structure of unautoprocessed form of IS1-inserted Pro-subtilisin E with Classification: Hydrolase from Organism *Bacillus subtilis* subsp. *subtilis* str. 168. Gene Names: *aprE*, *apr*, *aprA*, *sprE*, BSU10300 (EC: 3.4.21.62). Subtilisin catalyzes protein and peptide amides hydrolysis and is an extracellular alkaline serine protease (Graycar et al., 2013). Also, aprE gene of *Bacillus* sp. is reported to have strong fibrinolytic activity which strongly supports fibrinolytic attribute of our RFEA1 protein (Yao et al., 2017). However, in some cases proteins with similar folds might have distinct functions (Skolnick et al., 2015; Kinyanyi et al., 2018).

**Table 3:** TM-align identified top 10 structural analogs of RFEA1.

**Rank**^a^	**PDB hit**	**TM-score**	**RMSD**^b^	**IDEN**^c^	**Cov**^d^
1	3whiA	0.905	0.81	0.983	0.911
2	3afgB	0.857	2.11	0.346	0.911
3	1r6vA	0.847	3.04	0.331	0.942
4	2e1pA	0.831	1.93	0.434	0.877
5	4tr2A	0.829	2.90	0.246	0.932
6	3qfhA	0.802	2.43	0.260	0.874
7	6mw4A	0.788	2.86	0.249	0.884
8	1t1eA	0.749	3.56	0.176	0.887
9	3edyA	0.729	3.76	0.125	0.879
10	1lw6E	0.709	0.71	0.858	0.714

**Notes.**

a PDB protein structure ranking based on TM-score of structural alignment amid RFEA1 structure and known structures in PDB library.

b RMSD is root mean square deviation amid residues structurally aligned by TM-align.

c IDEN signifies percentage sequence identity in the structurally aligned region.

d Cov represents the alignment coverage and is equal to number of structurally aligned residues divided by length of the query protein sequence.

### Prediction of enzyme commission number and gene ontology terms of RFEA1

COFACTOR server predicted both Enzyme Commission Number (EC) and Gene Ontology term by structural comparisons (local and global) of RFEA1 model with proteins in BioLiP database (Roy, Yang & Zhang, 2012). The server suggested five Enzyme Commission (EC) numbers and active sites with Cscore^EC^ 0.618, 0.562, 0.557, 0.539 and 0.502. The model with highest and reliable Cscore^EC^ 0.618 based on PDB ID: 3bx1B with EC number 3.4.21.62 corresponds to enzyme subtilisin, a serine endopeptidase produced by various *Bacillus* species. Also, the predicted GO terminologies were used to infer modelled RFEA1 molecular and biological functional annotation. The consensus prediction of RFEA1 function suggests that it exhibits serine type-endopeptidase (GO: 0004252, GO score: 0.99) and proteolysis (GO: 0006508, GO score: 0.99) activity. The cellular component suggested extracellular region (GO: 0005576, GO score: 0.65). These findings support the serine endopeptidase and proteolytic attribute of RFEA1.

### In-silico interaction of RFEA1 with Fibrin

**Figure 4:**
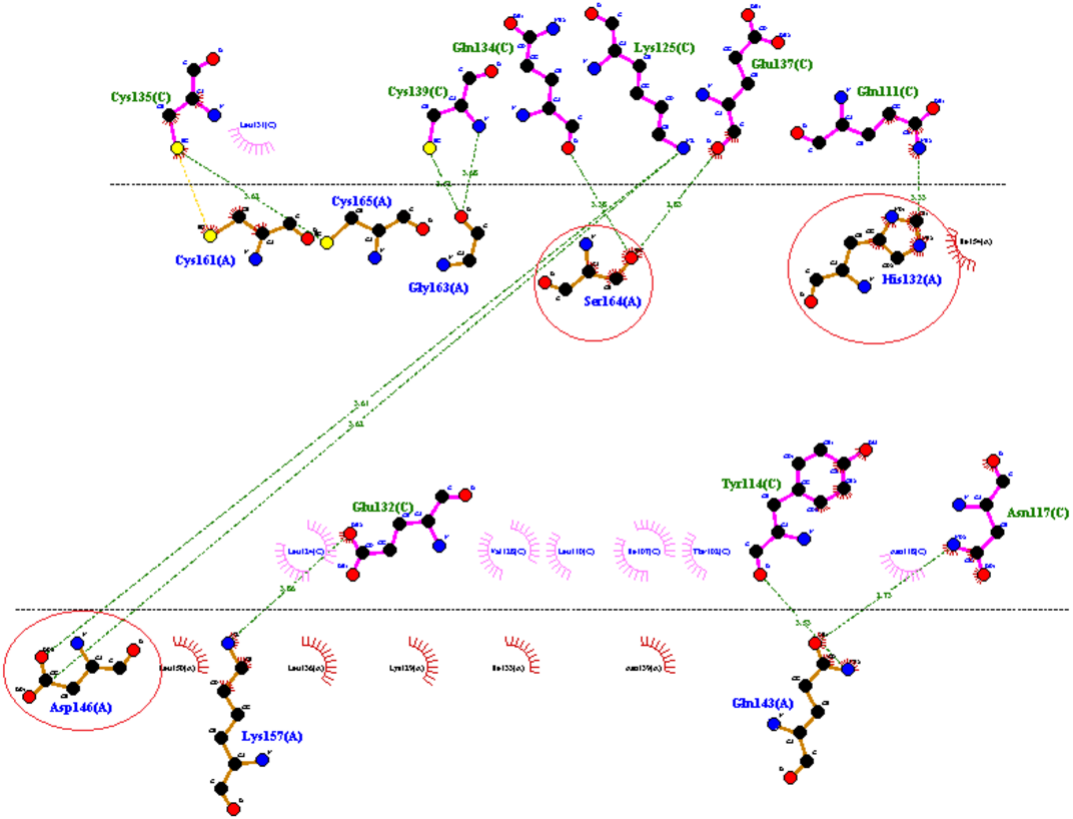
2D representation of interactive residues of RFEA1-fibrin complex using Ligplot. Encircled (red) are the catalytic triad residues of RFEA1 ‘Asp^146^, Ser^164^ and His^132^’.

PATCH DOCK server is a docking algorithm based on shape complementarity principles and was employed to study molecular interactions between fibrinolytic enzyme RFEA1 and fibrin. Figure S4 illustrates the details of output files of predicted RFEA1-fibrin interactive complexes. PATCH DOCK results were further refined and investigated for binding energy and hydrogen bonding in the complex with FIRE DOCK server. FIRE DOCK refined top 10 solutions (Fig. S5) and indicated that solution No. 7 of PATCH DOCK results has highest binding energy of −21.36 kcal/ mol with hydrogen bond contribution of −5.11. The binding energy between protein-protein complexes is demarcated by their contact region and interface, and determines their interaction strength. The more negative value of binding energy signifies stronger interaction between both the proteins. The 3D view of RFEA1-fibrin interaction was visualized using Chimera software (Fig. S6) and the interactive site residues of RFEA1 and fibrin were examined using Ligplot (Fig. 4). Total 9 amino acid residues of fibrin (Cys^135^, Cys^139^, Gln^134^, Lys^125^, Glu^137^, Gln^111^, Glu^132^, Tyr^114^ and Asn^117^) and 8 residues of RFEA1 (Cys^161^, Cys^165^, Gly^163^, Ser^164^, His^132^, Asp^146^, Lys^157^ and Gln^143^) served as hotspots which are involved in enzyme-substrate binding. The results thus showed that catalytic triad of RFEA1 ‘Asp^146^, His^132^, and Ser^164^’ was involved in interaction with fibrin residues ‘Lys^125^, Gln^111^, Gln^134^ and Glu^137^’. The In-silico results regarding binding affinity and substrate specificity of RFEA1 were then compared with the in-vitro results obtained in our previous study (Sharma et al., 2020).

### In-vitro validation

The in-vitro analysis revealed a gradual increase in fibrinolytic activity of RFEA1 in the presence of 0.5–2.00 mM of Ca^2+^ ions after 2 h of incubation (Table 4). An increase of approximately 30% in the activity of RFEA1 (130.87 ± 1.76%) was observed with 2 mM CaCl_2_. However, slight reduction in the activity (125.71 ± 1.82 and 119.98 ± 1.87%) was detected with 2.5 and 3.0 mM of CaCl_2_. Also, with 2 mM of CaCl_2_, a significant increase in the stability of RFEA1 was testified at different temperatures. An increase of 45.65, 41.25, 36.98, 30.17, 26.71, 21.12 and 10.58% in fibrinolytic activity was observed at temperature 20, 30, 40, 50, 60, 70 and 80 °C, respectively (Table 5).

Further, the in-vitro efficacy of RFEA1 has been evaluated using both fibrin and mammalian blood clot as substrate in our previous study (Sharma et al., 2020). The study reported higher affinity of RFEA1 towards fibrin with *K*_m_ and *V*_max_ values of 1.093 mg/mL and 52.39 ug/mL/min. The thrombolytic potential of RFEA1 was evaluated in comparison to a commercial thrombolytic agent streptokinase/myokinase (Biocon, India), using mammalian blood clot. The endogenous fibrinolytic factors such as plasmin and plasminogen were deactivated by thermal treatment of blood clots. Complete clot dissolution was observed within 4 h with RFEA1 and streptokinase.

**Table 4:** Effect of varying concentration of CaCl_**2**_ on RFEA1 activity.

**CaCl**_**2**_**concentration (mM)**	**Fibrinolytic activity (%)**
Control	100
0.5	108.21 ± 1.14
1.0	115.99 ± 1.32
1.5	123.01 ± 1.42
2.0	130.87 ± 1.76
2.5	125.71 ± 1.82
3.0	119.98 ± 1.87

**Table 5:** Effect of CaCl_**2**_ on fibrinolytic activity of RFEA1 at different temperatures.

**Temperature (°C)**	**Control (%)**	**Activity of CaCl**_**2**_**treated samples (%)**
20	100	145.65 ± 1.27
30	95.98 ± 0.79	137.23 ± 1.04
40	91.23 ± 0.52	128.21 ± 1.09
50	88.46 ± 0.49	118.63 ± 0.93
60	81.01 ± 0.44	107.72 ± 0.81
70	77.89 ± 0.65	99.01 ± 0.57
80	74.64 ± 0.76	85.22 ± 0.78

### Identification of conserved motifs of RFEA1

**Figure 5:**
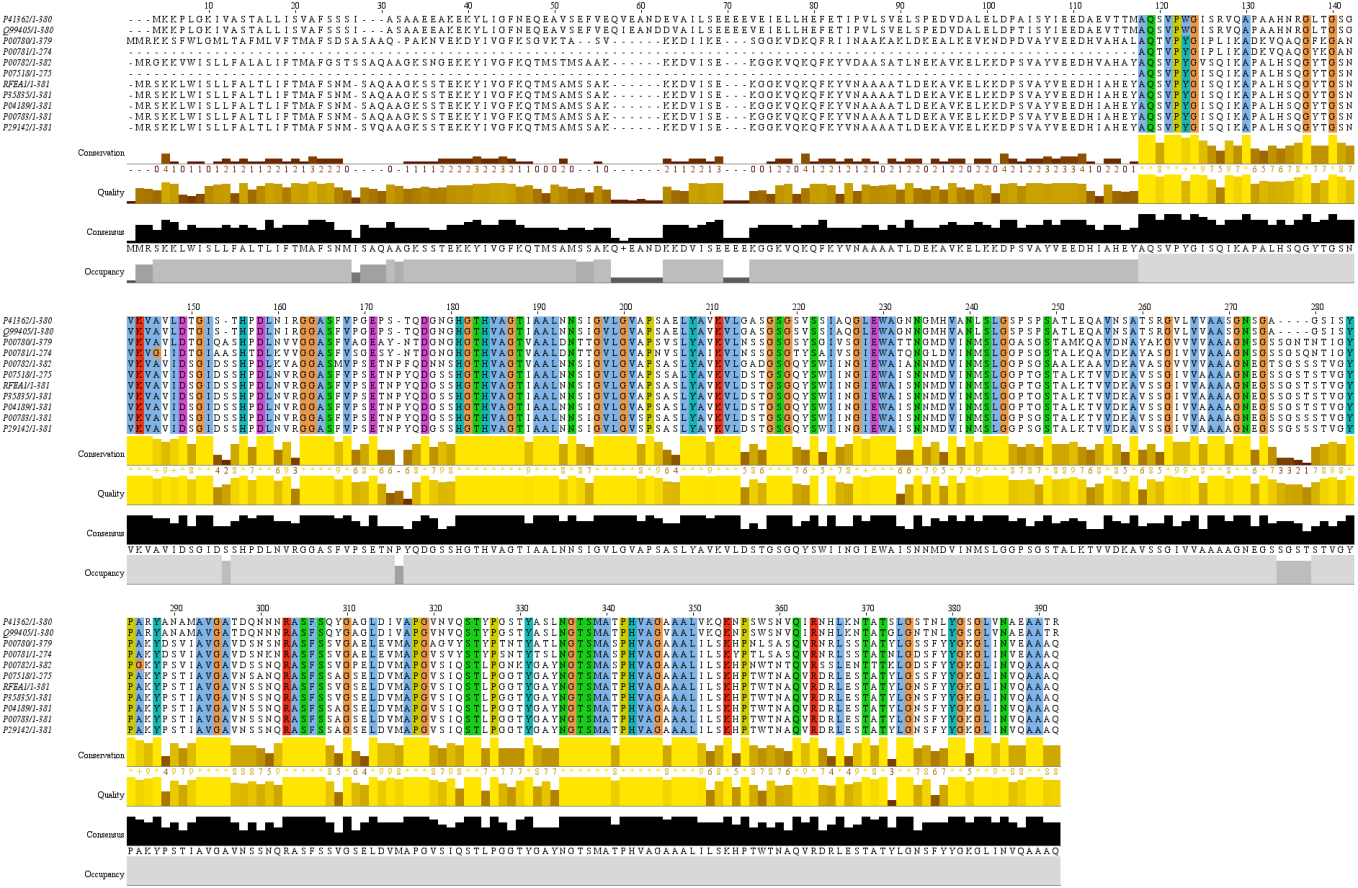
Multiple sequence alignment of RFEA1 with other homologous enzymes. Multiple sequence alignment with top 10 closest homologs of RFEA1. ELYA_BACCS P41362 Alkaline protease (3.4.21.-); PRTM_BACSK Q99405 M-protease (3.4.21.-); SUBC_BACLI P00780 Subtilisin Carlsberg ECO:0000303—PubMed:4967581; SUBD_BACLI P00781 Subtilisin DY (3.4.21.62); SUBT_BACAM P00782 Subtilisin BPN’ (3.4.21.62) (Alkaline protease); SUBT_BACPU P07518 Subtilisin (3.4.21.62) (Alkaline mesentericopeptidase); SUBN_BACNA P35835 Subtilisin NAT (3.4.21.62); SUBT_BACSU P04189 Subtilisin E (3.4.21.62); SUBT_BACSA P00783 Subtilisin amylosacchariticus (3.4.21.62); SUBT_GEOSE P29142 Subtilisin J (3.4.21.62). Coloured sections with an asterisk (^∗^) indicate the highly conserved amino acid residues.

**Figure 6:**
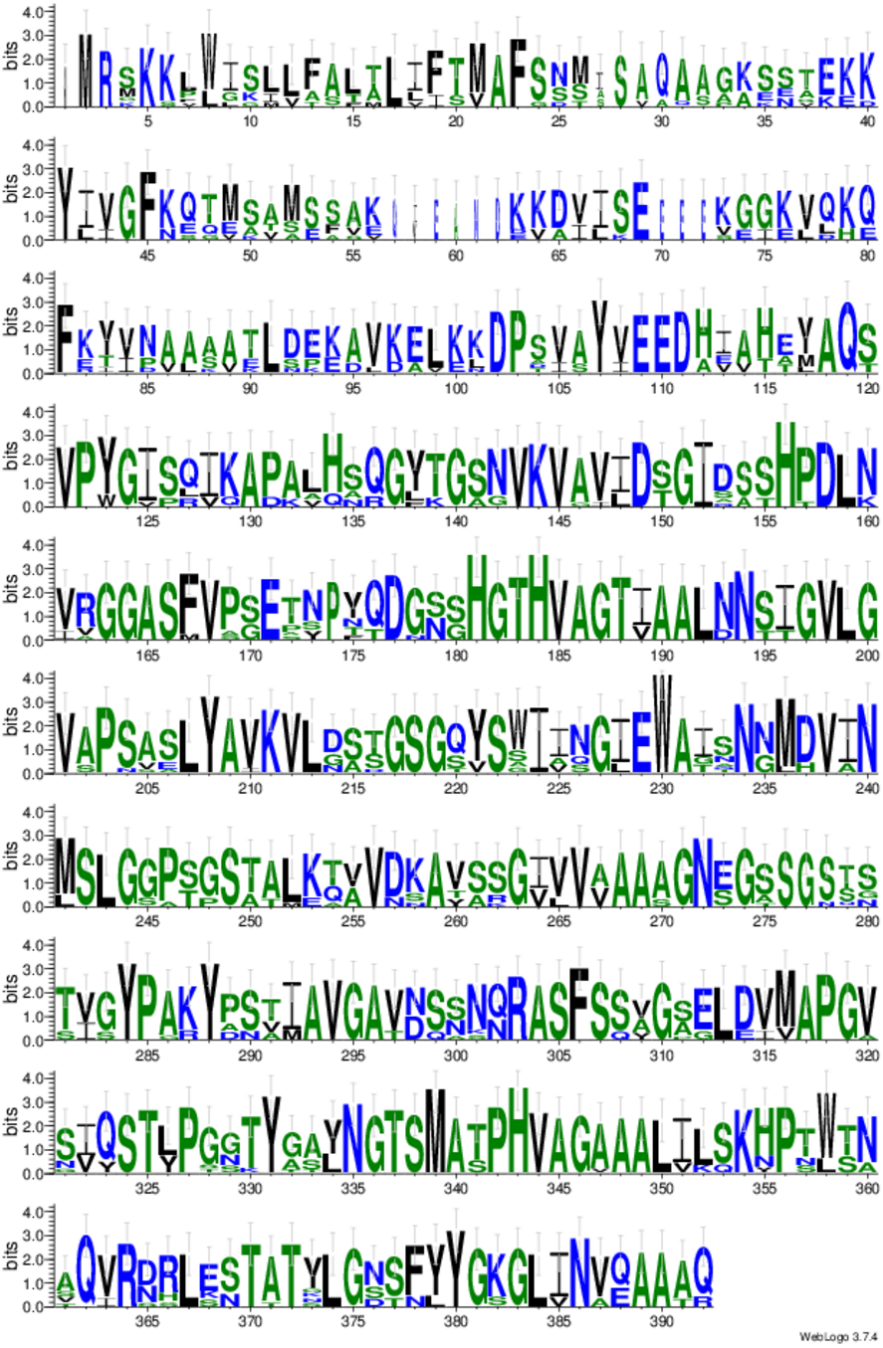
Sequence logo for conserved domain analysis of RFEA1 constructed using Weblogo. Each stack of symbol designates an amino acid residue. The color of stack is displayed according to the hydrophobicity of residues (hydrophilic residues–blue, neutral residues–green and hydrophobic residues–black). The overall height of stack indicates the degree of conservation whereas symbol height within the stack signifies relative frequency of each residue at that position. The sequence positions in the conserved domains are represented by numbers on the *x*-axis whereas *y*-axis denotes the information content estimated in bits.

Conserved domain analysis of RFEA1 was predicted using top 10 homologous enzymes, as depicted by PSI-BLAST, through multiple sequence alignment constructed using ClustalW (Fig. 5). UNIPROT: SUBN_BACNA P35835 Subtilisin NAT (3.4.21.62) (Nattokinase) (Precursor) with Length: 381, 100% Identity, 742 Score (bits), 100% Positives and *E*-value = 0 was observed to exhibit maximum homology with RFEA1 sequence. Furthermore, moderate similarity of RFEA1 sequence was examined with UNIPROT: SUBT_BACSU P04189 Subtilisin E (3.4.21.62) (Precursor); Length: 381, 99% Identity, 740 Score (bits), 99% Positives and *E*-value = 0. Multiple sequence alignment suggests numerous conserved columns with a score of 11 indicated by asterisk (^∗^) and mutations (Score:10) but conserved properties marked with plus (+). The analysis suggests that RFEA1 is a subtilisin-like fibrinolytic serine protease obtained from novel *Bacillus cereus* RSA1, with homologs from other sources as well. Besides, the sequence logos of homologous enzymes were generated using Weblogo server to further construct a clear alignment of the identified conserved domains. The results indicated that very low/no significant conservation was found at positions 1, 27, 57–62 and 70–72, whereas highly conserved domains were present at 181–188, 207–213, 303–307, 335–340 and 342–350 residue positions (Fig. 6).

## Discussion

Scientific studies suggest that limited attempts are made to explore the 3D structures and intermolecular interactions of bacterial fibrinolytic enzymes. In this investigation, we have focused on structural-functional analysis of fibrinolytic enzyme RFEA1 obtained from *Bacillus cereus* RSA1. Statistical interpretation, structural depiction and ligand interactions of RFEA1 has been successfully accomplished to get an insight into enzyme’s attributes. In-vitro validation and comparative analysis of the work is performed to confirm our In-silico predictions.

Statistical analysis by SAPS webserver has been efficiently used to predict precise composition of RFEA1. The server predicted RFEA1 as a serine (13.1%) and alanine (12.6%) rich protein. In *Mycobacterium tuberculosis* H_37_Rv, Rv3906c gene was testified to exhibit glycine (17.8%) and aspartate (23.7%) rich residues using SAPS server (Beg, Thakur & Meena, 2018). The use of multiple structural modeling servers is preferred for prediction of high-quality 3D protein models (Guleria et al., 2016; Lagares et al., 2020). We have used I-TASSER, SWISS-MODEL, RaptorX and Phyre2 to model RFEA1, wherein Phyre2 server predicted superior model with 99% identity and 100% confidence with template ‘3whiA’. The good quality and reliability of RFEA1 built model was confirmed using RC, ERRAT and Verify3D plots. Such validation parameters have been used in many scientific studies to achieve similar objectives (Beg, Thakur & Meena, 2018; Gupta et al., 2017; Manochitra & Parija, 2017). Further, model-template alignment suggests that RFEA1 exhibits huge dissimilarity in the position of residues and lacks a segment of 13 residues with respect to template (3whiA). Reports also suggest that homologous enzymes might lack few residues, in spite of high identity (Herrera-Zúñiga et al., 2019). COFACTOR - a significant tool used in many computational studies to analyse molecular/biological annotations of proteins (Beg, Thakur & Meena, 2018; Kinyanyi et al., 2018; Naveed et al., 2018), predicted RFEA1 as serine protease.

Our analysis further predicts one Ca^2+^ (Ca1) binding site in RFEA1, which is involved in regulation of enzyme’s fibrinolytic activity and thermostability. RFEA1 showed an increase of 30.87% in activity with enhanced thermal stability (45.65, 41.25, 36.98, 30.17, 26.71, 21.12 and 10.58%) at 20, 30, 40, 50, 60, 70 and 80 °C, in presence of CaCl_2_. Studies report that subtilisin enzymes from *Bacillus* sp. have two calcium binding sites (high-affinity Ca1 & low-affinity Ca2). Such bound Ca ions (specifically Ca1) have an important role in thermal stability and protection against autolysis. Ca1 site is highly conserved in several subtilisins and contributes to protein stabilization (Uehara et al., 2013; Smith et al., 1999). Fibrinolytic enzymes AprE176 and M179 from *Bacillus subtilis* HK176 and mutated *Bacillus subtilis* HK176 showed increased thermostability due to the presence of calcium binding sites. AprE176 retained 11% of its activity at 45 °C after 5 h whereas, M179 retained 36% (Jeong et al., 2015). Calcium-bound crystal structure of IS1-ProS221A (3whi) was fully folded and more stable than calcium-free form by 13.1 °C (Uehara et al., 2013). Fibrinolytic activity of enzyme from *Bacillus subtilis* DC27 was enhanced (122.02 ± 5.71%) in the presence of Ca^2+^ ions (5 mM) (Hu et al., 2019).

Further, In-silico enzyme-substrate interaction revealed that Lys^125^, Gln^111^, Gln^134^ and Glu^137^ residues of substrate (fibrin) interact with Asp^146^, His^132^, and Ser^164^ residues of RFEA1. When compared with literature, active sites Asp^19^, His^51^, and Ser^208^ of Subtilisin K2 from *Bacillus subtilis* K2 interacted with Leu^168^, Ile^171^, and Leu^172^ of the fibrin. Also, the binding energy of RFEA1 (−21.36 kcal/ mol) is more negative than Subtilisin K2 (−19.4 kcal/ mol) (Syahbanu et al., 2020), suggesting strong interaction between RFEA1 and fibrin. In-vitro results support our In-silico findings of high fibrin affinity of RFEA1 (*K*_m_: 1.093 mg/mL and *V*_max_: 52.39 ug/mL/min). In addition, RFEA1 has also been assessed for its thrombolytic potential using mammalian blood clot. The clot dissolution efficacy of RFEA1 (complete dissolution within 4 h) is high when compared to current thrombolytic enzymes (Sharma et al., 2020). The enzyme (0.2 and 0.5 µg) from *Schizophyllum commune* resulted in clot dissolution within 8 h (Lu & Chen, 2010) whereas orally administered nattokinase showed complete dissolution of blood clot within 5 h in a dog model (Yogesh & Halami, 2017). The docking and in-vitro study thus suggested high substrate binding affinity, specificity and thrombolytic potential of RFEA1 than already reported fibrinolytic enzymes.

We have performed multiple sequence alignment of RFEA1 with its sequence homologs using CLUSTALW and visualized it using Jalview software. The tool CLUSTALW is testified to produce biological alignments of different sequences by means of seeded guide trees and HMM profile-profile methods (Sievers & Higgins, 2018; Sievers et al., 2011; Dong et al., 2020; Saxena & Singh, 2015). Also, Jalview—a sequence alignment editing, visualisation and analysis tool is stated to yield significant results in numerous computer-aided sequence alignment examinations for diagnosing conserved residues (Kinyanyi et al., 2018; Waterhouse et al., 2009; Sanyanga & Tastan-Bishop, 2020). Lastly, sequence logos are created by WebLogo with graphical illustrations of the patterns within a multiple sequence alignment. Such logos provide significant and precise interpretation of sequence similarity than consensus sequences (Crooks et al., 2004) and are extensively used in numerous studies for identification of conserved motifs (Li et al., 2016; Vujaklija et al., 2016). RFEA1 alignment results revealed five highly conserved stretch of amino acids located in homologous regions of the aligned sequences. His^181^–Thr^188^ and Pro^342^–Leu^350^ are the largest identical domains among the aligned sequences followed by Leu^207^–Leu^213^, Arg^303^–Ser^307^ and Asn^335^ –Ala^340^. However, Ile^210^ of SUBD_BACLI P00781 Subtilisin DY and Val^347^ of PRTM_BACSK Q99405 M-protease are non-conserved residues among the specified domains. Jia et al. (2010) reported sequence alignment of propeptides from different subtilisins and indicated presence of three conserved regions (Tyr^10^–Lys^15^, Gly^34^ –Ala^46^ and Leu^59^ –Asp^71^). Another alignment study of a fibrin-degrading enzyme (subtilisin K2) from *Bacillus subtilis* K2 reports a highly conserved domain (Ala^1^–Gln^262^) (Syahbanu et al., 2020). Our study on the contrary reports some segments with no significant conserved residues: Met^1^ (SUBC_BACLI P00780 Subtilisin Carlsberg), Ile^27^ (ELYA_BACCS P41362 Alkaline protease and PRTM_BACSK Q99405 M-protease), Ala^27^ (SUBC_BACLI P00780 Subtilisin Carlsberg), Ser^27^ (SUBT_BACAM P00782 Subtilisin BPN), Gln^57^ (ELYA_BACCS P41362 Alkaline protease and PRTM_BACSK Q99405 M-protease), Val^58^ (ELYA_BACCS P41362 Alkaline protease), Ile^58^ (PRTM_BACSK Q99405 M-protease), Glu^59^, Ala^60^, Asn^61^, Asp^62^ and Glu^70^–Glu^72^ (ELYA_BACCS P41362 Alkaline protease and PRTM_BACSK Q99405 M-protease, respectively).

### Conclusion

Fibrinolytic enzyme RFEA1 from *Bacillus cereus* RSA1 with in-vitro thrombus hydrolysis potential might have tremendous possibilities towards industrial/therapeutic deployment in blood clot removal and treatment of cardiovascular thrombosis, respectively. Therefore, comprehending the structural attributes of RFEA1 is imperative to obtain further insights into its molecular and biological functional characteristics. Prediction of the in-silico 3D structural model is exceedingly challenging but beneficial for examination of structure-function aspects of a protein. The presented work is thus an effort to analyse structure based functional aspects of RFEA1. SAPS statistical compositional outcome has evidently presented RFEA1 as a serine (13.1%) and alanine (12.6%) rich protein with molecular weight 39.5 kDa. Validation statistics of modelled structure revealed that the Phyre2 server predicted the RFEA1 model better than other servers. Further study testified the presence of a high affinity calcium binding site in RFEA1. The in-silico molecular docking and in-vitro characterization reflects high binding affinity (−21.36 kcal/ mol) and substrate specificity of RFEA1 towards fibrin. Conclusively, this study provides an insight into structural functional characteristics of RFEA1 and might be a significant contribution in computational analysis for detection/identification of such fibrinolytic enzymes. Nevertheless, in-vitro analysis in our previous study (Sharma et al., 2020) and present study has reported similar characteristics of RFEA1, but in-vivo experimentations would be essential to confirm the claims.

##  Supplemental Information

10.7717/peerj.11570/supp-1 Supplemental Information 1 Model-Template alignment Marked (red) are the segments of 29 residues lacking in template with respect to RFEA1 and 13 residues lacking in RFEA1 with respect to template.

10.7717/peerj.11570/supp-2 Supplemental Information 2 ERRAT scores for the predicted four models of RFEA1 (A) I-TASSER modelled structure with overall quality factor 79.6247 (B) SWISS-MODEL modelled structure with overall quality factor 92.2156 (C) RaptorX modelled structure with overall quality factor 88.2038 (D) Phyre2 modelled structure with overall quality factor 89.645.

10.7717/peerj.11570/supp-3 Supplemental Information 3 Verify3D scores for the predicted four models of RFEA1 (A) I-TASSER modelled structure representing 88.98% of residues with averaged 3D-ID score > = 0.2. (B) SWISS-MODEL modelled structure representing 97.98% of residues with averaged 3D-ID score > = 0.2. (C) RAPTOR X modelled structure representing 88.19% of residues with averaged 3D-ID score > = 0.2. (D) Phyre 2 modelled structure representing 99.42% of residues with averaged 3D-ID score > = 0.2.

10.7717/peerj.11570/supp-4 Supplemental Information 4 Docking results of RFEA1 with fibrin using patchdock server Top 20 solutions are mentioned with docking score, area, ACE and transformation.

10.7717/peerj.11570/supp-5 Supplemental Information 5 Docking results of RFEA1 with fibrin refined using firedock server Rank 1 with solution number 7 is observed with highest global energy of −21.36 kcal/mol and hydrogen bonding of −5.11.

10.7717/peerj.11570/supp-6 Supplemental Information 6 3D view of RFEA1-fibrin docked complex visualized using Chimera software

## Additional Information and Declarations

### DNA Deposition

The following information was supplied regarding the deposition of DNA sequences: Sequences are available at NCBI: MK288105.

### Data Availability

The following information was supplied regarding data availability: All the data are available as Figures and Tables in the main article.
