# Supplementary figures and images for: Computational-approach understanding the structure-function prophecy of Fibrinolytic Protease RFEA1 from Bacillus cereus RSA1
Source: PeerJ. 2021 Jun 4;9:e11570. doi: 10.7717/peerj.11570 (PMC8183432; doi:10.7717/peerj.11570)

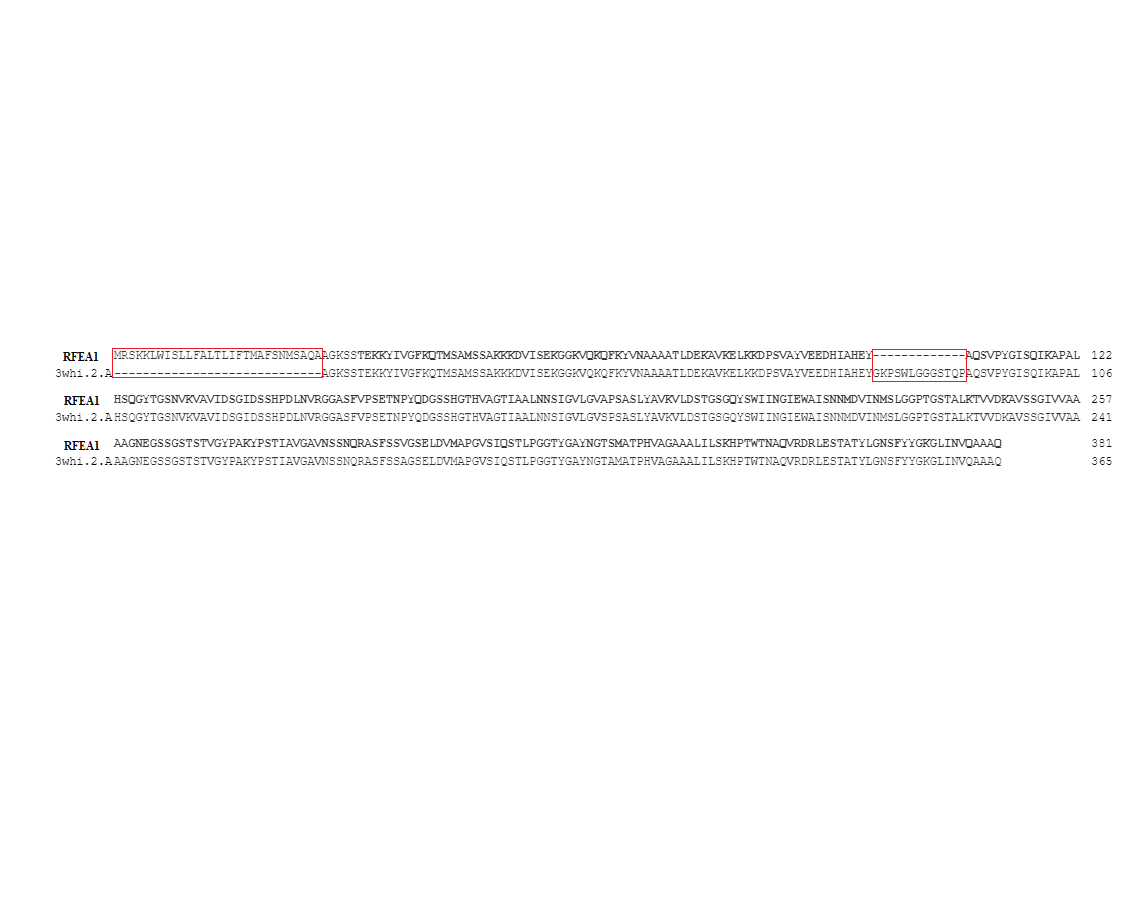

Supplement: Supplemental Information 1 — Marked (red) are the segments of 29 residues lacking in template with respect to RFEA1 and 13 residues lacking in RFEA1 with respect to template. [file peerj-09-11570-s001.png]

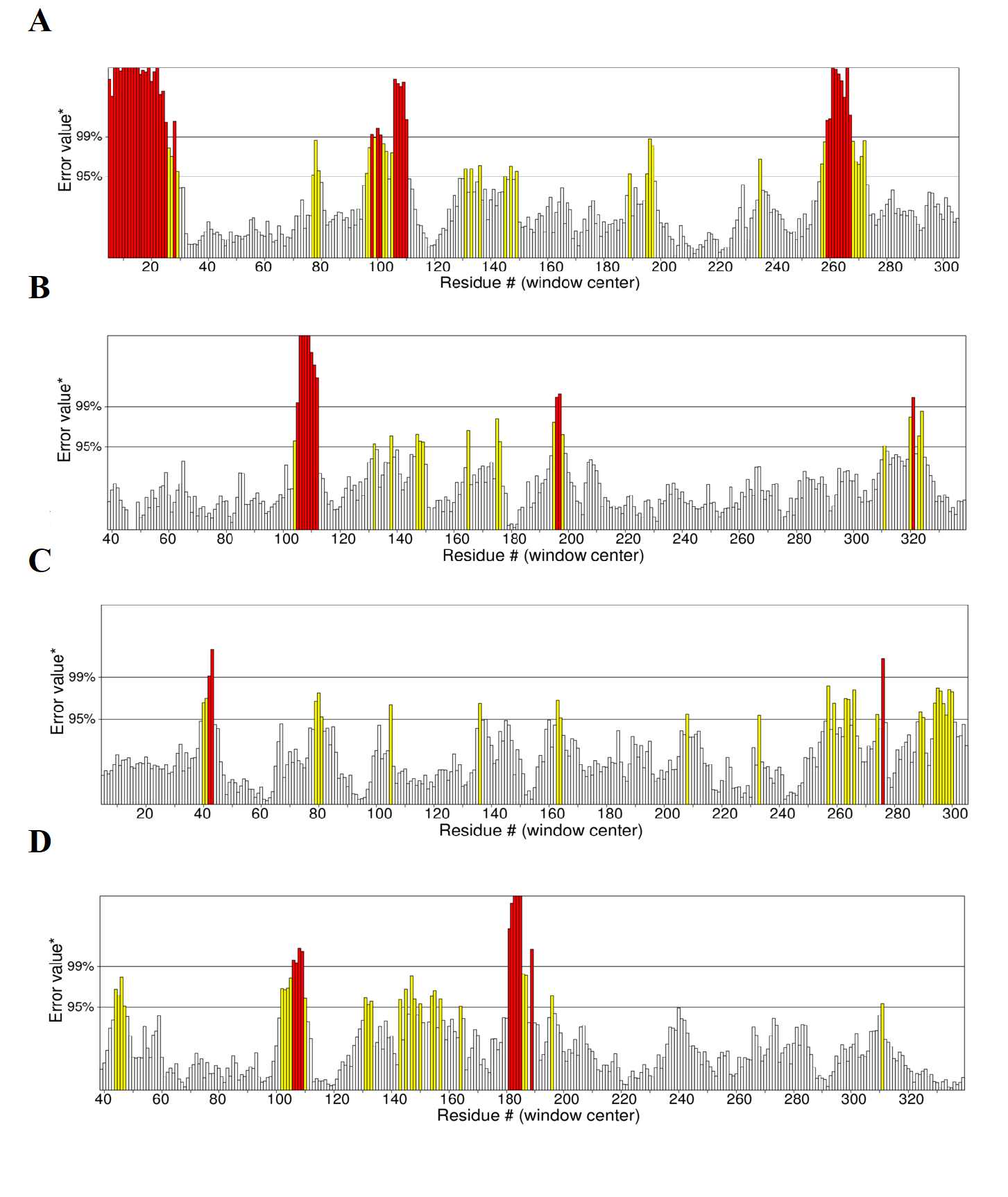

Supplement: Supplemental Information 2 — (A) I-TASSER modelled structure with overall quality factor 79.6247 (B) SWISS-MODEL modelled structure with overall quality factor 92.2156 (C) RaptorX modelled structure with overall quality factor 88.2038 (D) Phyre2 modelled structure with overall quality factor 89.645. [file peerj-09-11570-s002.png]

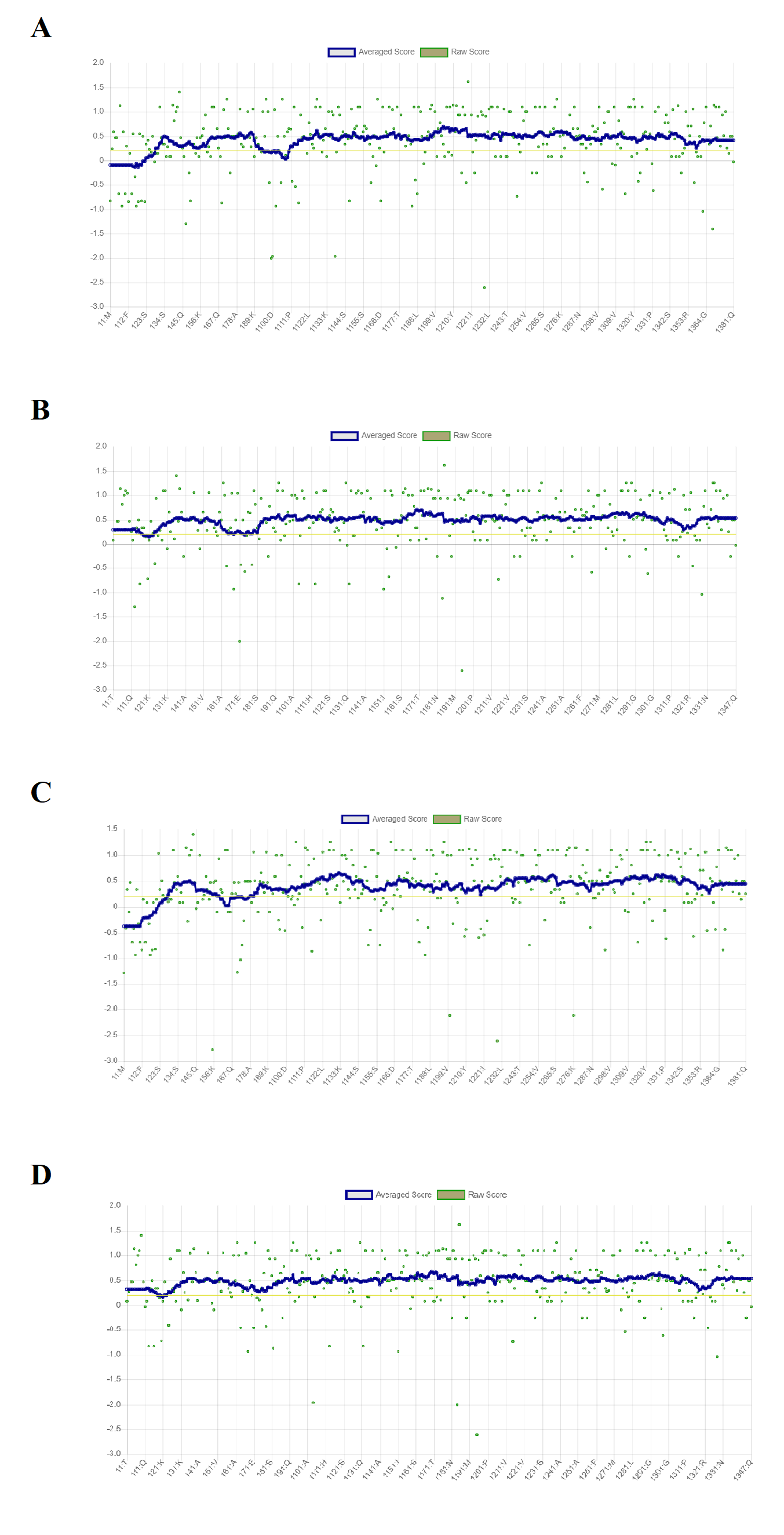

Supplement: Supplemental Information 3 — (A) I-TASSER modelled structure representing 88.98% of residues with averaged 3D-ID score > = 0.2. (B) SWISS-MODEL modelled structure representing 97.98% of residues with averaged 3D-ID score > = 0.2. (C) RAPTOR X modelled structure representing 88.19% of residues with averaged 3D-ID score > = 0.2. (D) Phyre 2 modelled structure representing 99.42% of residues with averaged 3D-ID score > = 0.2. [file peerj-09-11570-s003.png]

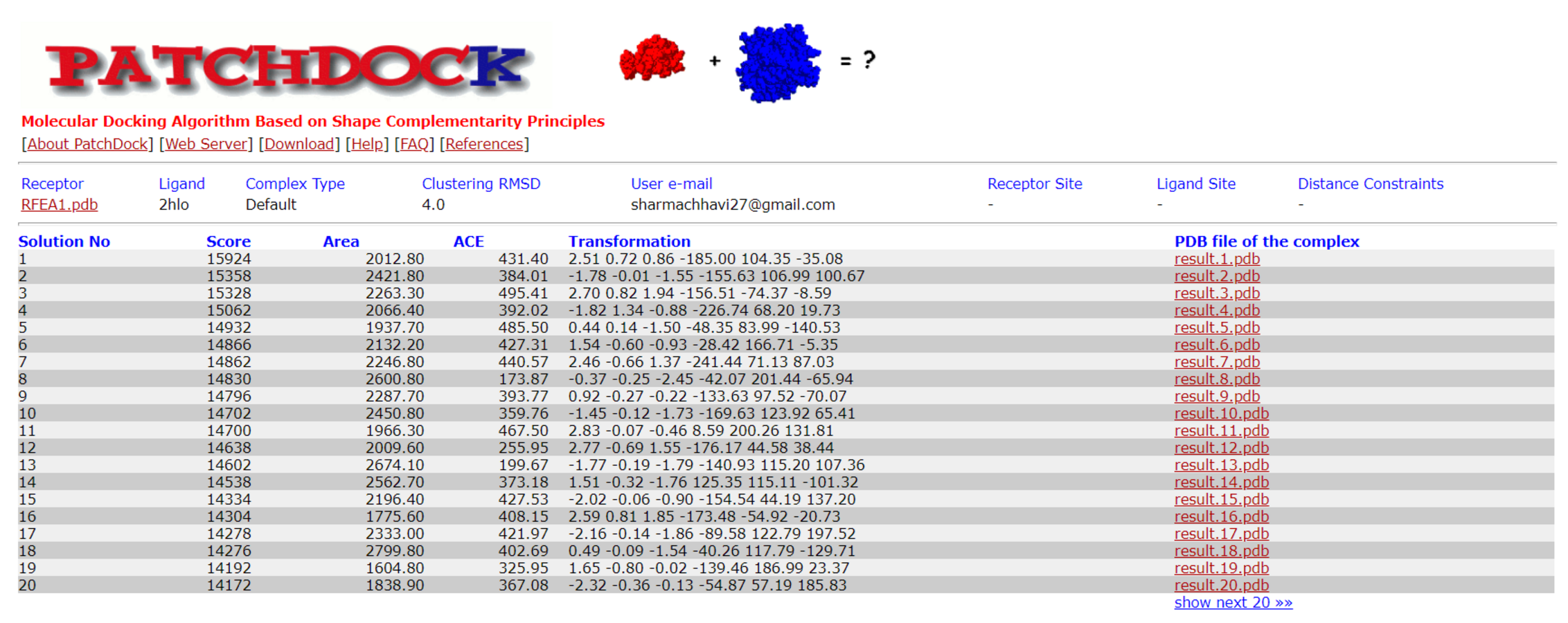

Supplement: Supplemental Information 4 — Top 20 solutions are mentioned with docking score, area, ACE and transformation. [file peerj-09-11570-s004.png]

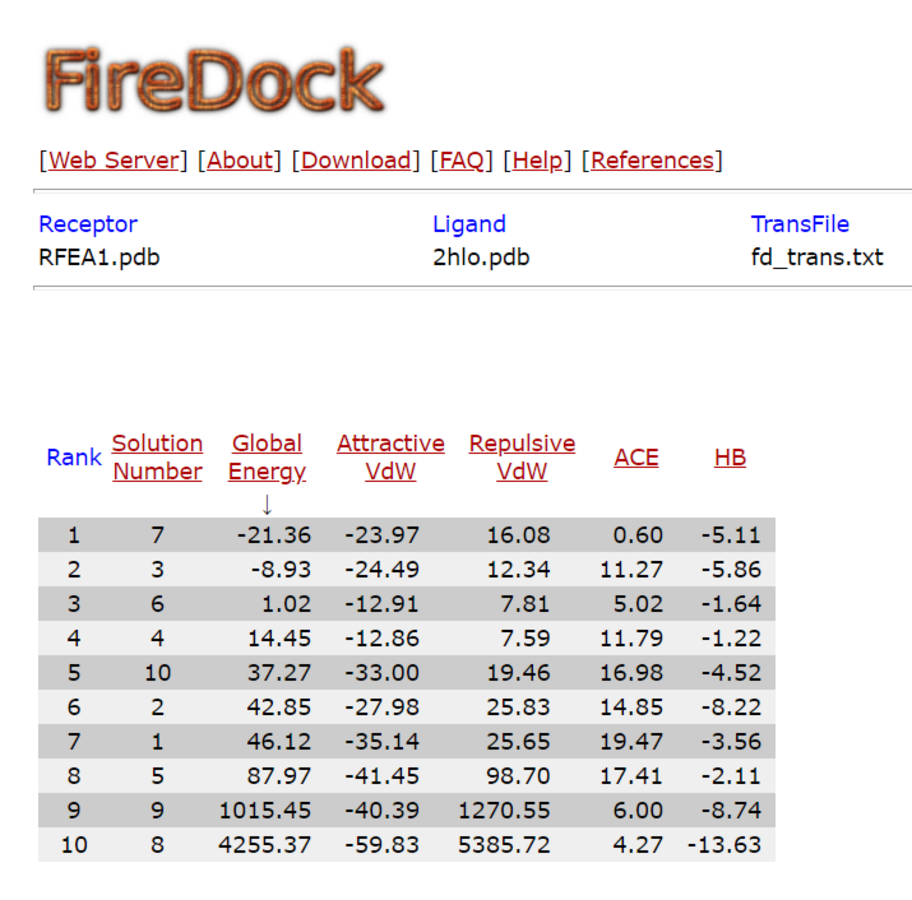

Supplement: Supplemental Information 5 — Rank 1 with solution number 7 is observed with highest global energy of −21.36 kcal/mol and hydrogen bonding of −5.11. [file peerj-09-11570-s005.png]

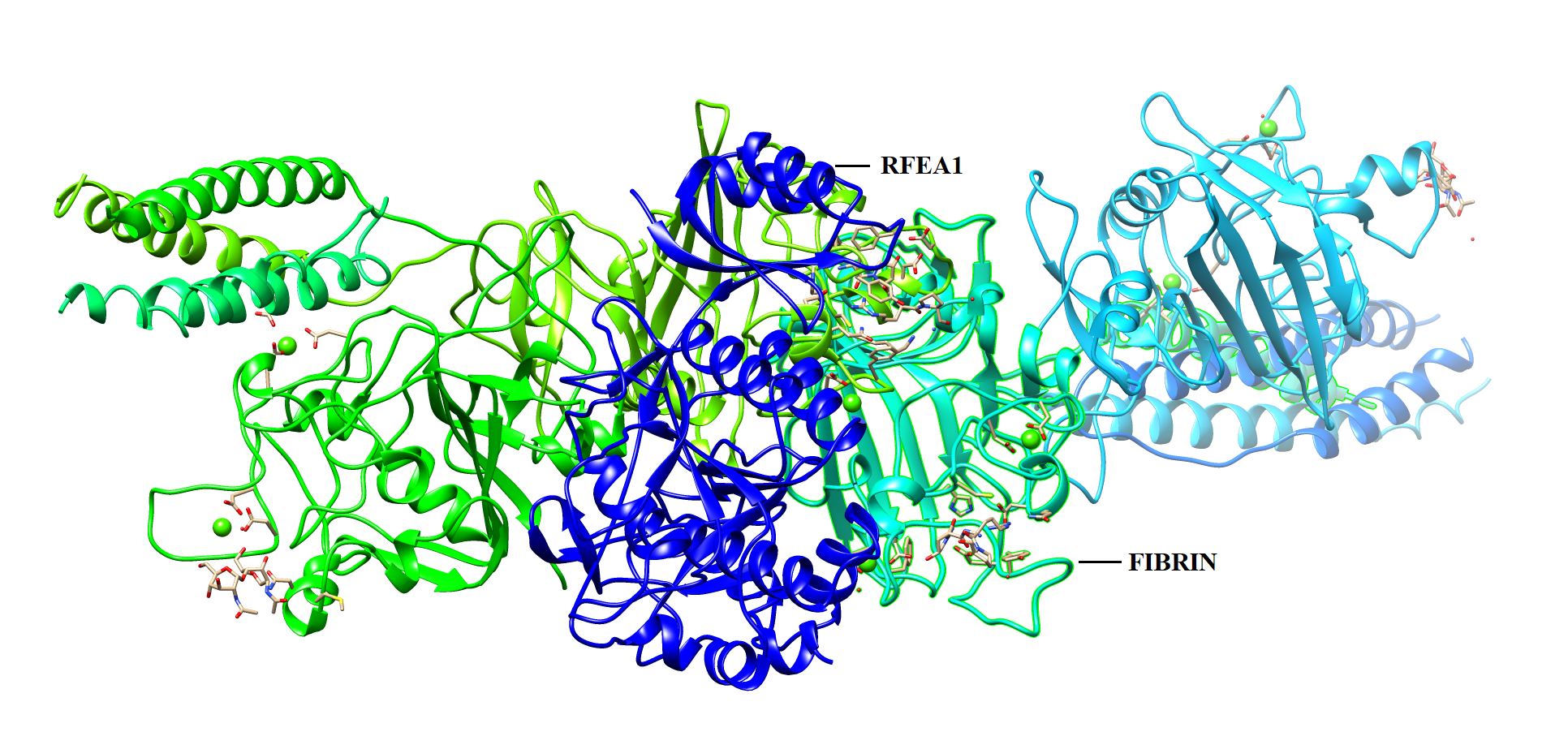

Supplement: Supplemental Information 6 [file peerj-09-11570-s006.png]
